# From Double Helix to Double Trouble: Sperm DNA Fragmentation Unveiled – A Reproductive Urologist Perspective (AUA Bruce Stewart Memorial Lecture – ASRM 2024)

**DOI:** 10.1590/S1677-5538.IBJU.2024.9924

**Published:** 2025-01-10

**Authors:** Sandro C. Esteves

**Affiliations:** 1 ANDROFERT Clínica de Andrologia e Reprodução Humana Campinas Brasil ANDROFERT, Clínica de Andrologia e Reprodução Humana, Campinas, Brasil; 2 Universidade Estadual de Campinas Departamento de Cirurgia Campinas Brasil Departamento de Cirurgia (Disciplina de Urologia), Universidade Estadual de Campinas (UNICAMP), Campinas, Brasil; 3 Aarhus University Faculty of Health Department of Clinical Medicine Aarhus Denmark Faculty of Health, Department of Clinical Medicine, Aarhus University, Aarhus, Denmark

## COMMENT

I was truly honored to have been nominated by the Society of Male Reproduction and Urology (SMRU), led by Dr. Kathleen Hwang, the current president, and Dr. Matt Coward, the president-elect, to give the prestigious American Urological Association (AUA) Bruce Stewart Memorial Lecture at the 2024 American Society for Reproductive Medicine (ASRM) Annual Meeting, held in Denver, Colorado.

This nomination holds special significance for me. I was fortunate to become a charter member of SMRU when it was founded nearly 30 years ago. At that time, I was in training at the Cleveland Clinic, mentored by one of the true giants in this field, Dr. Anthony Thomas Jr., right after completing my urology residency at UNICAMP with another esteemed mentor, Prof. Nelson Rodrigues Netto Jr. These early influences were foundational to my career, and to this day, my SMRU membership certificate holds a special place on my office wall. It was signed by Dr. Marc Goldstein, the first president of SMRU, a figure I have always held in the highest regard. One of my fondest memories dates back from an AUA meeting around that time, where Dr. Goldstein chaired a session alongside Dr. Craig Niederberger. It was during this session that I delivered my first-ever oral presentation at an AUA meeting, discussing my research on varicocele and azoospermia (1). Reflecting on that moment and witnessing the evolution of this field over the past three decades, it was a profound honor to present a lecture on sperm DNA fragmentation, an area in which our group has worked actively (2).

## INTRODUCTION

In the realm of male reproductive health, one pressing question persists: What if the greatest challenges we face are not rooted in a lack of knowledge but rather in the practical application of what we already understand?

This paper presents the author's expert opinion on sperm DNA fragmentation (SDF), a critical area where our understanding can directly influence reproductive success, and reflects the contents delivered during the lecture mentioned in the author's note above. The first part discusses the basics of sperm chromatin, its structural components, and the significance of protecting paternal DNA during sperm maturation and transport. The second part elaborates on the mechanisms of sperm DNA damage, the role of oxidative stress, and how SDF testing helps identify patients at risk. It includes specific tests used to measure SDF and their relevance in clinical settings. The last part discusses strategies to minimize SDF impact on male fertility and reproductive success, including lifestyle interventions, medical treatments (e.g., varicocelectomy), and the the use of testicular sperm in assisted reproductive technology (ART). This section highlights ours and other studies demonstrating the effectiveness of these interventions.

### Importance of Sperm DNA Fragmentation

In recent years, there has been a growing interest in studying human sperm chromatin, which comprises a complex mix of DNA and proteins. This structure holds not only genetic information but also crucial epigenetic signals necessary for creating healthy offspring (3). The proper packaging of sperm chromatin is vital to protect the paternal genome during its journey through the male and female reproductive systems, ensuring its delivery intact to the oocyte (4).

Sperm chromatin can suffer damage at various stages: during spermiogenesis, as it traverses the epididymis and even post-ejaculation. This damage can arise from multiple factors, including protamination failure, oxidative stress, and apoptosis. Notably, oxidative stress—primarily induced by high levels of reactive oxygen species (ROS)—significantly contributes to chromatin damage (5–7).

It is critical to recognize that sperm chromatin damage is a broad term that encompasses various structural defects. Sperm DNA fragmentation is a more specific term that refers to breaks in the DNA strands, which can be classified as single-strand or double-strand breaks (6, 8).

There is a robust association between oxidative stress and SDF, as human sperm are particularly vulnerable to free radical attacks (9, 10). These attacks compromise both the plasma membrane and the DNA within the sperm's nucleus and mitochondria, often leading to weakened DNA structures and strand breaks (6, 8).

**Figure 1:**
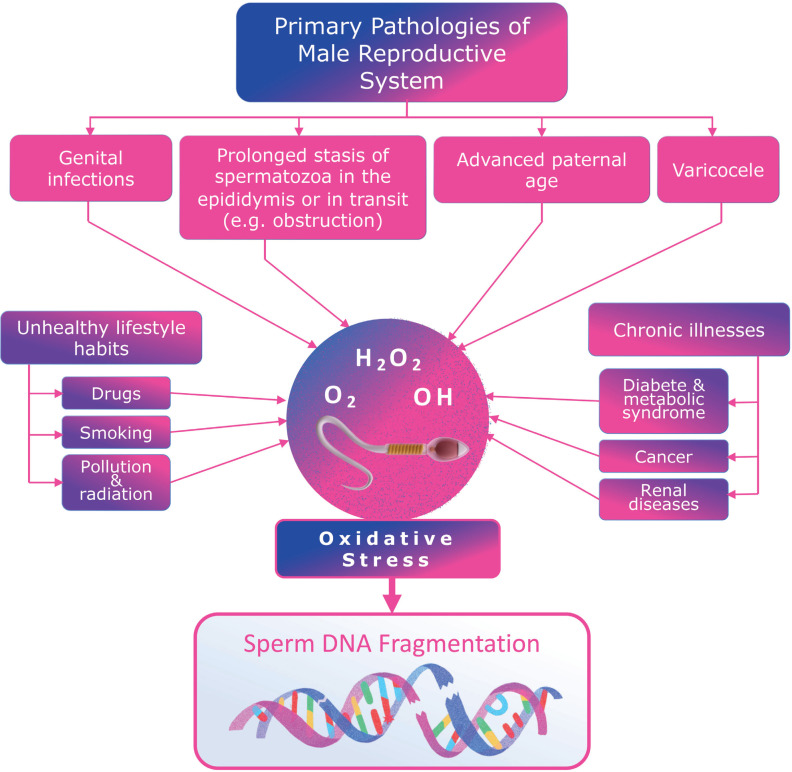
Clinical factors associated with increased oxidative stress in the male reproductive system that may contribute to sperm DNA fragmentation.

In clinical practice, various factors contribute to oxidative stress, including medical conditions like varicocele, genital infections, advanced paternal age, unhealthy lifestyle choices, chronic illnesses, and environmental toxins (4, 8, 11–13) (Figure-1). These factors promote an increased oxidative stress environment within the male reproductive system. Notably, SDF levels are consistently found to be higher in infertile men than in fertile controls and semen donors (14).

### Diagnosing and Managing Infertility through Sperm DNA Fragmentation Testing

**Figure 2:**
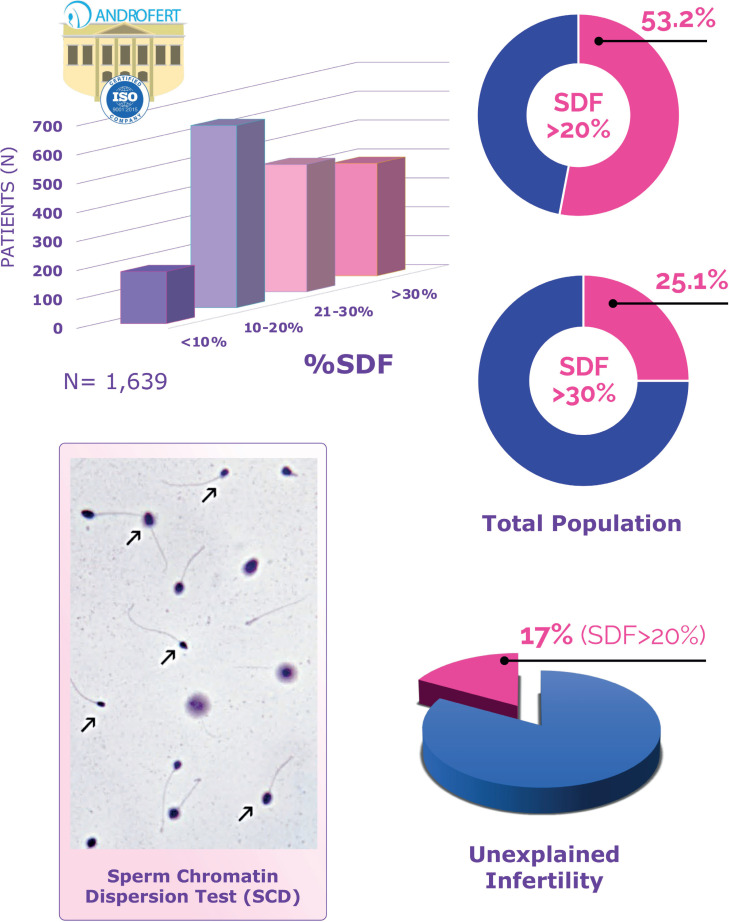
Prevalence of elevated sperm DNA fragmentation (SDF) among patients attending ANDROFERT, a tertiary center for reproductive medicine. The graph in the upper left quadrant illustrates the distribution of patients according to SDF levels. The graphs in the upper right quadrant show the proportion of patients with pathological SDF levels (above 20% on the top and above 30% on the bottom). The graph in the lower right quadrant depicts the proportion of patients with pathological SDF levels (i.e., >20%) among couples with unexplained infertility. The photomicrograph in the lower left quadrant displays the sperm chromatin dispersion test (SCD; Halo test), with arrows indicating individual spermatozoa lacking halos, signifying the presence of SDF (abnormal). In the central portion of the figure, two spermatozoa exhibit well-defined halos, indicating the absence of DNA fragmentation (normal).

In our clinic, where we routinely screen for SDF, more than 50% of patients exhibit rates exceeding 20%, which we consider the threshold indicating pathological SDF (15). Furthermore, around 25% of patients present with fragmentation rates above 30%, where the negative implications for reproductive outcomes become particularly pronounced (Figure-2).

Pathological SDF is commonly found in men with abnormal basic semen analysis parameters (16). However, it is also prevalent in male partners of couples facing unexplained infertility (8). For instance, a patient may present with basic semen analysis parameters within the reference ranges, have no apparent history of conditions affecting fertility, and display normal findings upon physical examination yet still possess pathological SDF that contributes to infertility (17).

Going back to oxidative stress, the hypothesis proposed by Professor Aitken and his research team from Australia presents an intriguing explanation of how oxidative stress leads to DNA fragmentation in human sperm (18). They suggest that the genesis of the problem begins during the late stages of spermatogenesis, where defective sperm with weakened chromatin are produced. These sperm, characterized by fragile DNA, become highly susceptible to oxidative attacks from both exogenous and endogenous sources, particularly hydrogen peroxide originating from the mitochondria. This process often results in DNA strand breaks that can be identified through specific laboratory tests.

### Measurement of Sperm DNA Fragmentation

**Figure 3:**
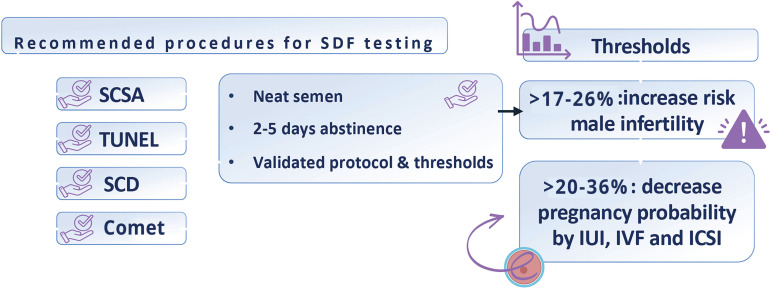
Key technical aspects of sperm DNA fragmentation (SDF) testing. SDF should be measured using SCSA, TUNEL, SCD, or alkaline Comet following established and validated protocols. The analysis must be conducted on neat semen collected after an abstinence period of 2 to 5 days, ideally between 2 and 3 days. All four methods (SCSA, TUNEL, SCD, and alkaline Comet) provide valuable insights into infertility risk and the likelihood of reproductive success. Abbreviations: TUNEL: terminal deoxynucleotidyl transferase-mediated dUTP-biotin nick end labeling, SCSA: sperm chromatin structure assay, SCD: sperm chromatin dispersion, Comet: single-cell gel electrophoresis, IUI: intrauterine insemination, IVF: in vitro fertilization, ICSI: intracytoplasmic sperm injection.

Sperm DNA fragmentation can be quantified using several well-established tests, including the TUNEL assay, the sperm chromatin structure assay (SCSA), the sperm chromatin dispersion test (known as the Halo test), and the alkaline Comet assay (6, 8, 19–21) (Figure-3). These methods can be categorized into two main types: those that utilize enzymatic reactions to label DNA breaks, such as TUNEL, and those that employ controlled DNA denaturation to reveal breaks, like SCSA, SCD, and Comet assays. While these tests measure the overall SDF level in a sample, they do not specify whether the breaks are in single or double DNA strands.

One critical point is that because each test employs a different method to detect DNA breaks, results obtained from one assay may not always align with those from another. Nevertheless, there is a strong correlation between the results from SCSA, TUNEL, alkaline Comet, and SCD tests when it comes to classifying patients as having normal or pathological sperm DNA fragmentation levels (22). Overall, research indicates a high level of agreement between laboratories concerning SDF measurement (23, 24).

Additionally, SDF levels in consecutive ejaculates display low biological variability. In a recent study, we evaluated the reliability of the SCD test for measuring SDF, specifically concerning the consistency of results obtained from the same patient at different time points (25). For this, we analyzed two semen samples collected from the same individuals, with a three-month interval between collections. We found that 80% of the patients remained in the same classification—either normal or pathological SDF across both analyses. The results demonstrated a high intraclass correlation coefficient, reflecting strong agreement between the two assessments, with only a minimal difference in SDF rates between the samples. Based on these results, we concluded that a single analysis is generally sufficient to assess SDF levels for most patients. However, for individuals with borderline levels, we recommend considering a confirmatory test, particularly when making treatment decisions. It is also worth mentioning that ejaculatory abstinence has a notable or pathological SDF; longer abstinence periods are associated with increased levels of SDF (26, 27).

Regarding SDF level thresholds, values exceeding the 17 to 26% range —dependent on the assay used—indicate an increased risk for male infertility (8, 19). Similarly, levels surpassing the 20 to 36% range are associated with an increased risk of adverse pregnancy outcomes, particularly in assisted reproduction scenarios (8, 19). While these cutoffs are informative, it is crucial to acknowledge that they are not infallible, especially when predicting pregnancy outcomes. The predictive value of SDF testing is influenced by the fertility status of the female partner, which warrants further discussion. There is yet to be a universally accepted gold-standard test for SDF. Each method has unique characteristics and may exhibit distinct clinical thresholds depending on the measured outcomes. Therefore, clinicians considering incorporating SDF testing into clinical practice should select the method that aligns best with their specific circumstances, taking into account factors like test availability, turnaround time to obtain reports and costs. Furthermore, identifying optimal thresholds tailored to their patient population should be also considered.

When conducting SDF testing, several key considerations must be remembered (Figure-3). First, SDF should be measured using one of the four validated methods previously discussed. It is essential to adhere to established protocols to ensure the accuracy and reliability of the results. Second, for accurate diagnosis and treatment planning, the analysis must be performed on the neat semen collected after a recommended abstinence period of 2 to 5 days, with an optimal duration of 2 to 3 days to minimize the risk of false positives. Third, it is critical to maintain a consistent abstinence period when the test is employed to monitor the effects of treatments aimed at reducing SDF. This consistency helps ensure the reliability of the results. Lastly, when performed correctly, all four methods can provide valuable insights into infertility risk and the likelihood of reproductive success, aiding in informed clinical decision-making. A detailed discussion about the technical aspects of SDF testing can be found elsewhere (8).

At Androfert, the SCD test (i.e., the Halo test) is used to assess SDF. We advise patients to maintain an ejaculatory abstinence period of 2 to 3 days before providing a semen specimen to ensure optimal test accuracy (8). Each test is conducted with positive and negative controls, and we always perform a basic semen analysis concurrently with SDF testing. We employ a cutoff of 20% to differentiate between normal and pathological SDF, with values exceeding 30% categorized as especially high (Figure-2).

### Negative Impact of Sperm DNA Fragmentation on Reproductive Success

The potential adverse effects of SDF on human reproduction are significant and warrant careful consideration. To fully understand these implications and to accurately interpret the existing literature, it is essential to revisit the underlying pathophysiology, particularly the hypothesis proposed by Aitken and colleagues (18). They suggest that oxidative stress can lead to the formation of base adducts, such as 8-oxo-deoxyguanosine, indicative of DNA damage.

**Figure 4:**
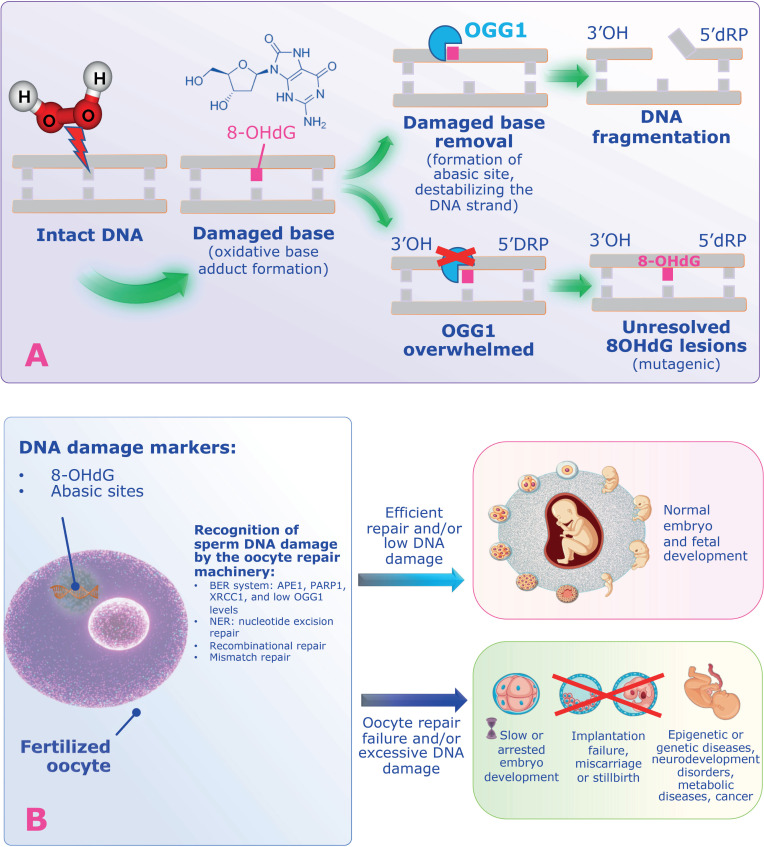
The Aitken and De Iuliis hypothesis for the origin of DNA fragmentation in human sperm. A) Free radicals’ attack, especially from hydrogen peroxide, can lead to the formation of base adducts, such as 8-oxo-deoxyguanosine, indicative of DNA damage. Sperm utilize the 8-oxo guanine glycosylase (OGG1) to repair these damaged bases. This process creates an abasic site that destabilizes the DNA strand and increases the risk of strand breaks. Excessive oxidative stress can overwhelm OGG1, resulting in persistent DNA lesions (e.g., 8-OHdG) that may cause mutations. B) As sperm lack the mechanisms for complete DNA repair, they rely on the oocyte's cellular machinery to further repair sperm DNA damage post-fertilization and before syngamy. If the oocyte fails to repair this damage adequately, the zygote may undergo a non-apoptotic mechanism that impairs paternal DNA replication. This impairment can lead to adverse outcomes, including poor embryo development, implantation failure, miscarriage, and an increased risk of congenital disabilities in the offspring. Abbreviations: OGG1: 8-oxi guanine glycosylase, 8-Oxo-dG: 8-oxo-deoxyguanosine, APE1: DNA (apurinic/apyrimidinic site) endonuclease 1, 5'dRP: 5-terminal deoxyribose phosphate, BER: Base excision repair, APE1: DNA (apurinic/apyrimidinic site) endonuclease 1, PARP1: poly (ADP-ribose) polymerase-1, XRCC1: x-ray cross-complementing protein.

To repair the damaged bases, sperm utilize the enzyme 8-oxoguanine glycosylase (OGG1), which removes the oxidized base. However, this repair process results in an abasic site destabilizing the DNA strand, increasing the likelihood of strand breaks. When oxidative stress is excessive, OGG1 can become overwhelmed, leaving persistent lesions on the DNA that may cause mutations (18) (Figure-4).

Unfortunately, sperm lack the necessary tools for complete DNA repair. Instead, they depend on the oocyte's cellular machinery to fix sperm DNA damage following fertilization and before syngamy (28). If the oocyte is unable to adequately repair the inflicted DNA damage, the resulting zygote may respond through a non-apoptotic mechanism that slow down paternal DNA replication (28). This impairment can lead to adverse outcomes such as poor embryo development, implantation failure, miscarriage, and an increased risk of congenital disabilities in the offspring—some of which may not manifest until future generations (28, 29).

The most substantial evidence linking SDF to reproductive outcomes is derived from animal studies, which often experience fewer confounding variables than human studies. For instance, a groundbreaking investigation by Yanagimachi and colleagues demonstrated that sperm DNA integrity deteriorates during epididymal transit (30). In a mouse model utilizing these defective sperm with abnormal chromatin for ICSI, there was a notable increase in chromosomal abnormalities within embryos and a corresponding decrease in both implantation and live birth rates (30). Additional research found that inducing oxidative DNA damage in epididymal sperm was associated with higher miscarriage rates and developmental defects in mouse offspring (31).

Translating these findings to human studies, elevated SDF has been associated with extended timeframes to achieve natural conception (32). Meta-analyses reveal that couples experiencing recurrent pregnancy loss (RPL) exhibit significantly higher SDF levels than fertile couples (33, 34).

Regarding the impact of SDF on IUI outcomes, meta-analyses consistently indicate that the risk of pregnancy failure more than doubles when sperm from men with elevated SDF levels are used (35, 36). Furthermore, when assessing IVF and ICSI outcomes, our group's recent review of existing meta-analyses found that while two analyses reported minimal negative impacts of SDF on pregnancy rates, eight identified significant negative effects on conventional IVF outcomes without affecting ICSI results (37). Additionally, two studies reported significant negative consequences in both conventional IVF and ICSI. Along these lines, when it comes to miscarriage rates in IVF/ICSI pregnancies, the association remains consistent; high SDF levels are associated with an elevated risk of miscarriage in both conventional IVF and ICSI procedures (38–40).

While it is essential to critically evaluate the current knowledge and acknowledge that the evidence linking SDF to adverse effects on human fertility is not entirely conclusive, we must also adopt a clinical perspective. The impact of SDF on reproductive outcomes primarily hinges on the interplay between the severity of DNA damage and the oocyte's capacity to repair it effectively. Unfortunately, many of our patients present with factors such as advanced maternal age or diminished ovarian reserve. When these issues coincide with elevated SDF levels, the potential implications for fertility become particularly concerning.

### Holistic Approaches to SDF Testing

Given the robust association between SDF and male infertility—and the potential detrimental effects on reproductive outcomes—many experts now consider SDF analysis a frontline diagnostic procedure. Our clinical guidelines advocate for testing in specific scenarios, including cases of varicocele, unexplained and idiopathic infertility, recurrent miscarriage, assisted conception, fertility counseling, particularly when there are known risk factors for high oxidative stress, and when freezing sperm for fertility preservation (8). The results obtained from these tests can play a pivotal role in guiding management decisions.

The latest WHO semen analysis manual has also acknowledged the significance of SDF testing, incorporating it into the extended semen examination panel (41). This panel comprises advanced tests that may be utilized in clinical practice at the laboratory's discretion or upon the clinician's request.

Despite the growing recognition of SDF testing, it is essential to note that its role as a frontline diagnostic tool remains contentious. The 2021 ASRM/AUA male infertility guidelines, recently updated, do not endorse SDF testing as a routine component of the initial infertility evaluation for couples (42, 43). However, they do suggest it for couples with a history of recurrent pregnancy loss (RPL). Conversely, the updated 2024 European Association of Urology (EAU) guidelines take a broader approach by strongly recommending SDF testing for couples with RPL, whether resulting from natural conception or ART (44, 45). They also advise testing for men with unexplained infertility.

The strong correlation between high SDF levels and RPL has led authoritative organizations, such as the European Society for Human Reproduction and Embryology (ESHRE) and the Australasian Reproductive Endocrinology and Infertility Consensus Expert Panel, to include SDF testing in their evaluations for couples experiencing RPL (46, 47). The Australasian guidelines have even introduced an algorithm to assist clinicians in treatment decisions based on SDF results (47).

The key guidelines’ statements and recommendations concerning SDF testing are summarized in Table-1.

**Table 1:** Guidelines’ recommendations for sperm DNA fragmentation testing.

Guideline [Year]	Statement	Grade of Recommendation/ Level of evidence
SFRAG guidelines [2021]	Situations for considering SDF testing:	Ranging from conditional^1^to strong^2^recommendation; evidence level^3^ranging from B to D
	Varicocele Unexplained and idiopathic infertility Pregnancy loss, especially when recurrent When assisted conception is contemplated For fertility counseling, particularly when there are risk factors for high oxidative stress When freezing sperm for fertility preservation	
WHO semen analysis manual [2021]	SDF is an extended semen examination (advanced test) that may be used clinically in certain situations* by choice of the laboratory or at the special request of the clinician.	NA
AUA/ASRM male infertility guidelines [2021; updated 2024]	SDF analysis is not recommended in the initial evaluation of the infertile couple; For couples with recurrent pregnancy loss, men should be evaluated with SDF.	Moderate recommendation; evidence level C
EAU guidelines on sexual and reproductive health [2021; updated 2024]	Perform SDF testing in the assessment of couples with recurrent pregnancy loss from natural conception and failure of ART or men with unexplained infertility.	Strong recommendation; evidence level 2a
ESHRE guidelines on recurrent pregnancy loss [2018; updated 2022]	Assessing SDF in couples with recurrent pregnancy loss could be considered for diagnostic purposes.	Conditional
Australasian recurrent pregnancy loss clinical management guideline [2024]	SDF testing is suggested to evaluate the contribution of the male factor in recurrent pregnancy loss. The guideline proposes management strategies in the presence of pathological SDF levels, including lifestyle modifications, assessment of varicocele, use of antioxidants, and IVF with advanced sperm selection techniques.	Evidence levels ranging from 1 to 3

* Not specified;

NA = not applicable; SDF = sperm DNA fragmentation; ART = assisted reproductive technology; WHO: World Heath Organization; AUA: American Urological Association; ASRM: American Society for Reproductive Medicine; EAU: European Association of Urology; ESHRE: European Society for Human Reproduction and Embryology.

While the AUA/ASRM guidelines’ cautious stance against the routine use of SDF testing is understandable—primarily due to the limited evidence supporting its predictive value for pregnancy—it is vital to recognize that SDF is not solely a matter of fertility. There is a burgeoning concern regarding the potential health implications of high SDF levels for the resulting offspring. If SDF is not entirely repaired in the oocyte, it may lead to genetic or epigenetic mutations in the embryo, which could have long-term effects on the child's health (29). These concerns include potential alterations in cardiometabolic health, neurodevelopmental disorders, and even childhood cancers, which may extend into future generations (29, 48).

We now understand that most *de novo* mutations in our species originate from the paternal genome, often arising from defective DNA damage repair mechanisms (49, 50). In a recent article, I posited that while ART, particularly ICSI, can enable couples to conceive without addressing the underlying causes of male infertility, it is not without risks, especially when using sperm from men with elevated SDF levels (51).

New evidence continues to emerge regarding the negative effects of high SDF. A recent study examining birth outcomes from IVF and ICSI, utilizing data from the Swedish National Registry, found a strong association between SDF and adverse events such as preterm birth and preeclampsia (52). These detrimental outcomes are recognized as potentially influenced by paternal factors, given that the placenta is genetically derived from both the mother and the father. However, until now, the precise mechanisms underlying these associations have remained elusive. This study underscores the critical role that SDF may play in these outcomes.

### Strategies to Minimize SDF Impact

**Figure 5:**
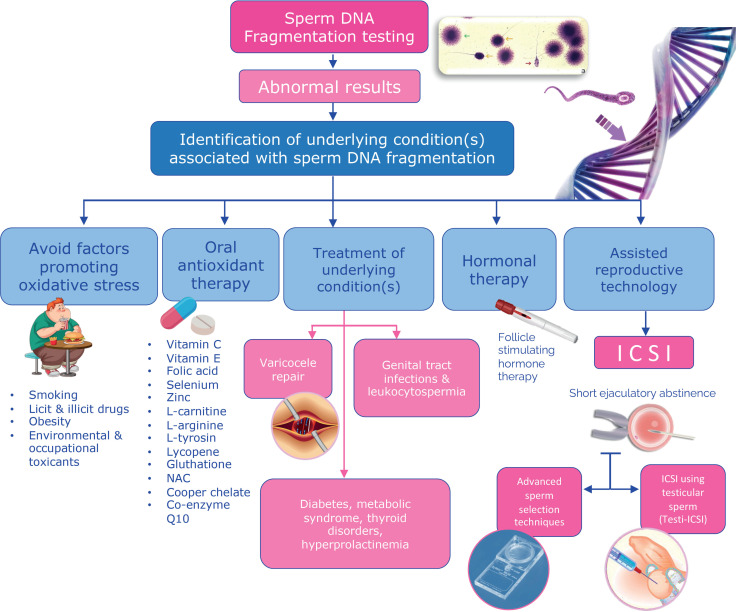
Clinical and laboratory strategies to mitigate the effects of pathological sperm DNA fragmentation of male fertility and human reproduction. A comprehensive male evaluation by a qualified reproductive urologist or andrologist is crucial for identifying and addressing conditions associated with poor sperm DNA quality. Several interventions have been explored to reduce SDF rates, including lifestyle modifications, oral antioxidant supplementation, varicocele repair, treatment of clinical and subclinical male genital infections, and exogenous FSH therapy. Additional strategies in the context of assisted conception include frequent ejaculations, short ejaculatory abstinence, advanced sperm preparation techniques (e.g., hyaluronic acid and microfluidics), the preference for intracytoplasmic sperm injection (ICSI) over intrauterine insemination and conventional in vitro fertilization (IVF), and the use of testicular sperm for ICSI.

From the extensive body of evidence we have discussed, I strongly advocate including SDF analysis as an integral component of best clinical practices to benefit our patients and their children. Identifying pathological SDF is essential, as it opens avenues for therapeutic interventions to reduce fragmentation levels (Figure-5).

Numerous interventions have been explored in this context (15). Notably, the insights gained from measuring SDF levels are most valuable when integrated with a comprehensive male evaluation conducted by a qualified reproductive urologist or andrologist. This evaluation should encompass a thorough medical history, physical examination, and any necessary diagnostic tests to identify and potentially address conditions adversely affecting sperm DNA quality or to optimize ART usage (53, 54).

For example, in a prospective single-arm pilot study, we investigated the effects of a three-month lifestyle intervention combined with daily antioxidant intake (55). Following the guidelines set forth by the Danish Health Authority, participants were instructed to reduce red meat consumption, increase their intake of fruits and vegetables, limit sugary beverages and alcohol, and engage in daily exercise. Additionally, the intervention included commercially available oral antioxidants. The study included couples with a history of unsuccessful IVF/ICSI attempts where the male partners had pathological SDF levels. We compared the changes in SDF post-intervention with those in a control group that did not undergo the intervention. The results demonstrated a mean reduction in levels of approximately 7 percentage points after the intervention, compared to only a 0.4% change in the controls. These preliminary findings suggest that lifestyle modifications combined with antioxidant supplementation can effectively lower SDF levels.

Furthermore, varicocele management is another crucial intervention area, given its association with oxidative stress and SDF (11, 12, 56). In our systematic review and meta-analysis, which encompassed 19 studies, we examined the effects of varicocelectomy on SDF levels (57). The findings indicated that treating a clinical varicocele significantly reduced SDF levels, with an average relative decrease of 30% from baseline. Additionally, a meta-regression analysis revealed that this reduction was more pronounced in men with higher baseline SFD levels, particularly those exceeding 20%.

In the study mentioned above, we also analyzed pregnancy outcomes, and found that postoperative SDF levels were significantly lower in patients from couples who achieved pregnancy than those who did not (57). This underscores the potential for reducing SDF to enhance pregnancy rates in men with clinical varicoceles. To address a clinical varicocele, we employ microsurgical techniques, utilizing intraoperative Doppler ultrasound to improve precision during the procedure (58–60).

In the context of ART, simple measures such as encouraging frequent ejaculations and ensuring that the patient provides a semen sample on the day of oocyte retrieval—following a short abstinence period of as little as one day—can make a significant difference (61, 62). These straightforward steps may help improve sperm DNA quality and the likelihood of successful outcomes in ART.

Another promising avenue for men with high SDF undergoing ICSI involves the use of testicular sperm (63–66). This approach may be advantageous due to the significantly lower levels of DNA damage present in testicular sperm compared to those that have undergone the typical journey through the epididymis, vas deferens, and ejaculate (67, 68). The critical factor seems to be related to avoiding oxidative stress encountered by sperm as they traverse the male reproductive system and following ejaculation (65, 69).

In a prospective observational study involving 172 couples with male partners diagnosed with idiopathic oligozoospermia and high SDF, we found that utilizing testicular sperm for ICSI, as opposed to ejaculated sperm, yielded significant improvements in outcomes (70). Specifically, using testicular sperm reduced the rates of miscarriage and increased the chances of live birth. Notably, we determined that for every five couples requiring testicular sperm, one additional successful live delivery was obtained. Moreover, when comparing SDF levels between testicular and ejaculated sperm within the same patients, we found that testicular sperm exhibited an approximately 80% reduction in fragmentation levels (70). This remarkable finding underscores the importance of minimizing oxidative stress during the maturation and transport of sperm.

This vital knowledge has been highlighted in the 2024 updated ASRM/AUA male infertility guidelines for the first time, underscoring the importance of considering SDF in clinical practice (43). According to the guidelines, clinicians may consider the utilization of testicular sperm in nonazoospermic males with elevated SDF index’.

Preliminary evidence also suggests that advanced laboratory techniques, such as using hyaluronic acid and microfluidics, hold promise for isolating sperm with lower DNA fragmentation levels for assisted conception (71, 72). However, it is crucial to recognize that even if these innovative techniques are validated to improve outcomes, they should never replace comprehensive male evaluations. Neglecting thorough assessments would mean missing unique opportunities to identify and treat underlying conditions that may adversely affect sperm DNA quality.

## CONCLUSIONS

It is essential to highlight several key takeaways regarding SDF and its impact on male infertility. First, high SDF is a significant factor that can increase the risk of infertility and adverse outcomes in IUI and ART, particularly when combined with factors such as advanced female age or poor oocyte quality. Therefore, it is imperative to consider all these factors to gain a complete understanding of the implications of SDF on reproductive success. Second, infertility is inherently a couple's issue. Relying solely on the assessments of one partner provides an incomplete picture. A holistic approach that includes evaluations of both partners is essential for effectively addressing infertility. Third, while SDF testing offers valuable insights into gamete quality, it should not be considered a substitute for basic semen analysis or comprehensive andrological evaluations. Instead, it represents one crucial component of a multifaceted approach to reproductive health. Finally, incorporating SDF testing into ART clinics is not just a good idea; it is an essential aspect of good clinical practice. This approach improves the care we provide and ensures that we offer our patients the most comprehensive and effective treatments possible.

To close, there are compelling reasons to amplify our focus on male infertility care. Our objective should be to achieve a balance in the attention already given to female infertility, ensuring optimal outcomes for the couples we serve. Achieving this goal will require fertility clinics to prioritize interdisciplinary collaboration with reproductive urologists and andrologists. Together, we can deliver the most thorough and effective care to our patients, ultimately enhancing their chances of successful reproduction.
